# Tools to measure membrane potential of neurons

**DOI:** 10.1016/j.bj.2022.05.007

**Published:** 2022-06-03

**Authors:** Anjul Khadria

**Affiliations:** Department of Chemistry, Chemistry Research Laboratory, University of Oxford, Oxford, UK

**Keywords:** Action potential, Membrane potential, Neurons, Voltage-sensitive dyes, Brain

## Abstract

The brain is the most unexplored part of our body. The lack of sufficient tools has hindered our understanding of the brain and the associated diseases. The study of neurons and the neuronal network will help elucidate how the brain functions and related disorders. Over the last few decades, an increasing number of techniques have been reported to study neurons and neuronal communication *in vitro*, *ex vivo*, and *in vivo*. These methods have pushed the boundaries of neuroscience and elucidated more information than ever before; however, much more requires to be done to understand the brain in its entirety. In this review article, I discuss the principles and the advantages and disadvantages of the classical electrode-based recording techniques and the optical imaging-based methods, which have aided neuroscientists in understanding neuronal communication.

It is believed that an adult human brain consists of over 100 billion neurons and a similar number of glial cells packed in a volume of about 1300 cm^3^ weighing approximately 1.5 kg and robustly protected by a skull and the formidable blood–brain barrier [1,2]. Since the neuron doctrine at the end of the nineteenth century [3,4]^,^ scientists have been exploring the brain and understanding how neurons communicate with each other and the rest of the body. It is well-known that the neurons use electrical signals *via* potential differences (membrane potential) developed across their plasma membranes for communication; however, very little is known about how these electrical signals help the brain and body function [[5], [6], [7]]. The membrane potential is generated by the concentration gradient-based movement of the sodium, potassium, and chloride ions between the nerve cell and the environment through the various ion-specific channels located in the plasma membrane [6]. In the eighteenth and nineteenth centuries, Galvani and Bernstein first studied the flow of electric current through nerve cells [[8], [9], [10]]; however, the most significant progress came in the last few decades after the development of several non-optical and optical techniques to measure the membrane potential in neurons with high spatiotemporal resolution [7,11,12].

## Membrane potential

**Fig. 1:**
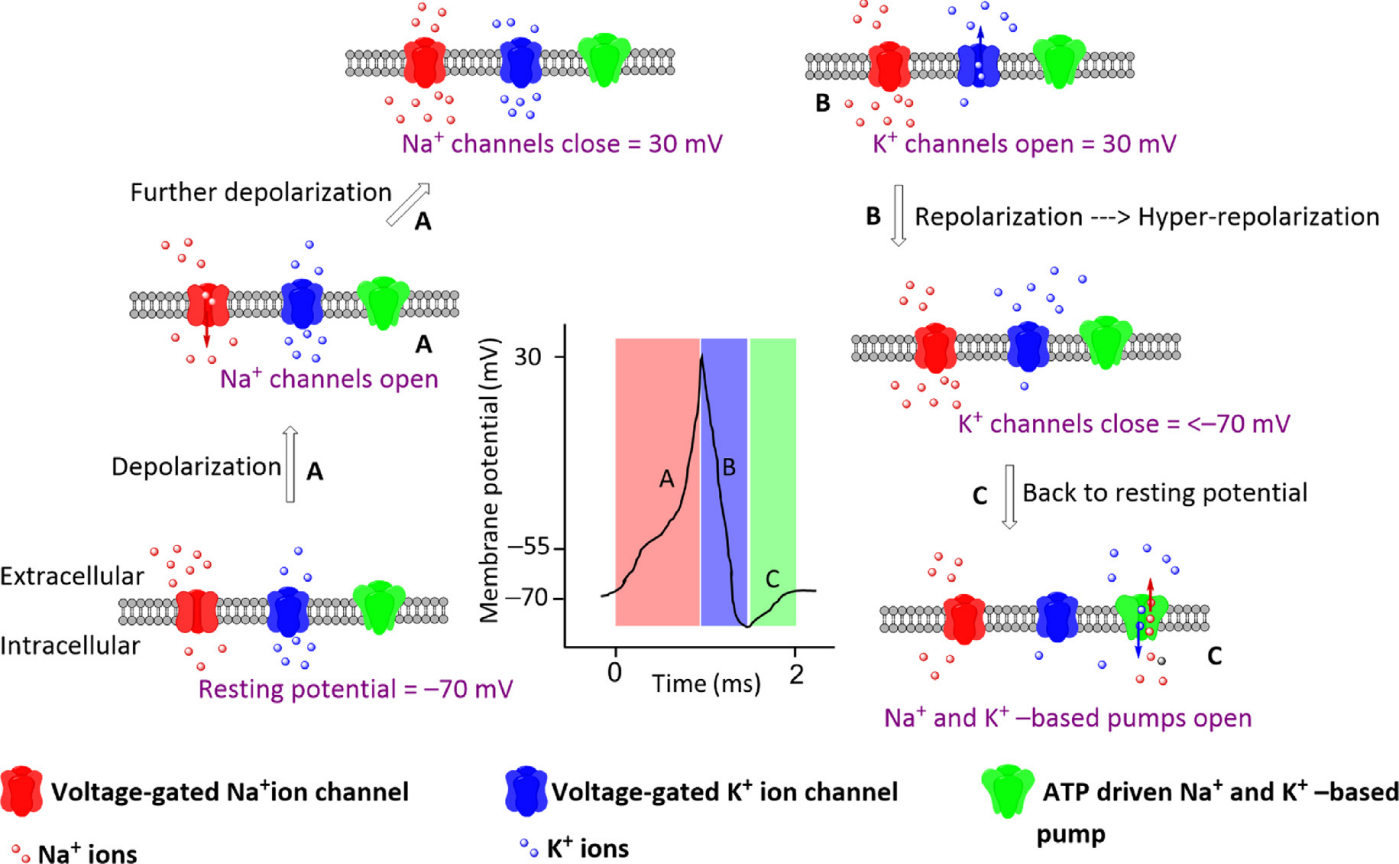
Depiction of activity of the ion channels in plasma membrane on the generation of an action potential.

When the potential difference across its plasma membrane is −70 mV, a neuron is at rest, known as the resting potential. At resting potential, there are more positively charged sodium ions outside the cells than inside and more potassium ions inside the cells than outside, leading to a concentration gradient. At the start of an action potential (initiated by a stimulus or generated spontaneously), the voltage-gated sodium channels open, leading to passive transport of positively charged sodium ions from outside the cells to inside, thus shifting the transmembrane potential towards a positive value. As soon as the voltage reaches a threshold value of −55 mV, more voltage-gated sodium ion channels open, depolarizing the neuron to a transmembrane potential of 30 mV. After depolarization, the voltage-gated sodium ion channels close, and the voltage-gated potassium ion channels open, causing the potassium ions to move from inside the cells to the outside, following which the cell repolarizes to resting potential [Fig. 1]. During repolarization, the voltage-gated potassium ion channels remain open for a longer duration causing hyper-repolarization leading to further decrease of transmembrane potential below −70 mV. Gradually, the voltage-gated potassium ion channels close, and an ATP-driven sodium and potassium –based pump opens to reinstate the cell at a resting potential of −70 mV [Fig. 1]. The whole cycle of depolarization and repolarization takes place in a time scale of about 2 ms and is called the ‘action potential’ [13]. In 1963, the Nobel Prize for Physiology or Medicine was awarded to John Eccles, Alan Lloyd Hodgkin, and Andrew Fielding Huxley for their work on nerve impulse transmission in terms of the action potential [14].

## Measurement of membrane potential

Several non-optical and optical –based techniques have been developed over the last few decades to measure the membrane potential of neurons. Here, we discuss the methods that have been developed over the years to study the membrane potential of neurons.

### Non-optical techniques

Non-optical techniques such as patch-clamp [15], intracellular recording [15], microelectrode arrays [16], and brain mesh electronics [17] have emerged as powerful tools to study the membrane potential/current of excitable cells.

#### Patch-clamp technique

In the patch-clamp technique, a low resistance (2–10 MΩ) glass microelectrode is attached to the cellular plasma membrane, and then the required voltage or current is measured. Patch-clamp–based techniques are divided into several subtypes: whole-cell patch-clamp, inside-out patch-clamp, outside-out patch-clamp, cell-attached patch-clamp, and perforated patch-clamp [18]. Each technique is used for various types of recordings, depending on the requirement.

**Fig. 2:**
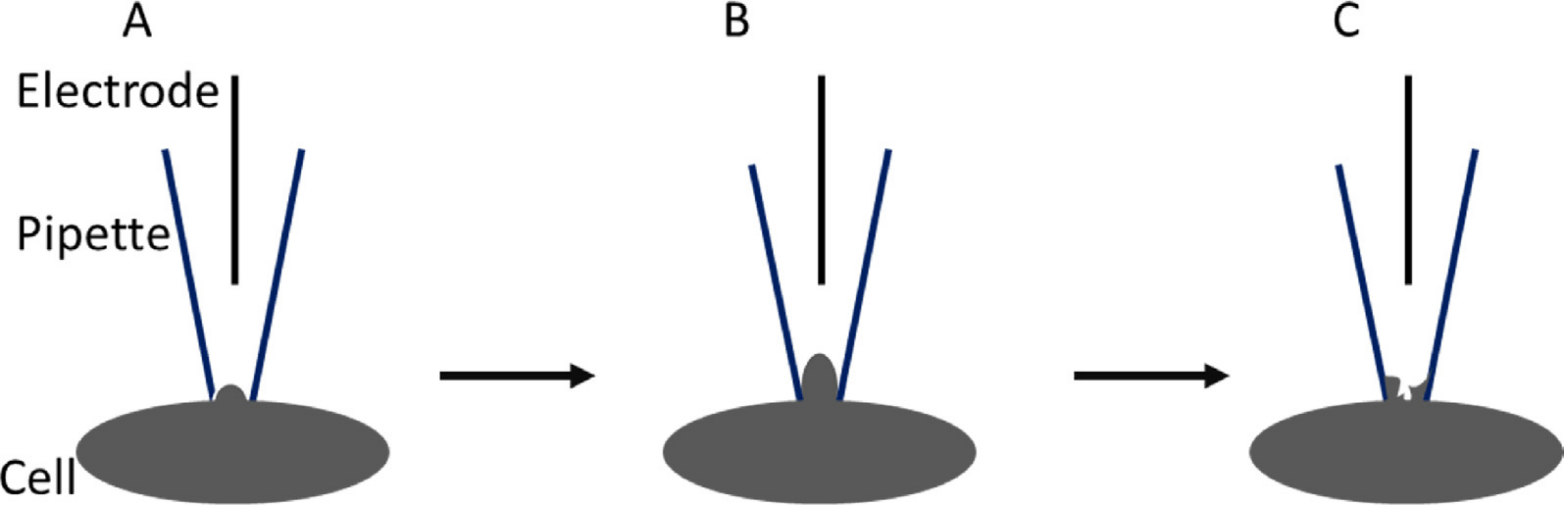
Steps to perform whole-cell patch-clamp. (A) An intracellular solution-filled glass pipette of tip resistance around 3–7 MΩ is gently pressed against the cell membrane without piercing through the membrane. (B) After making contact with the cell membrane, a mild suction is applied to aspirate a membrane patch in the glass pipette to form a seal of resistance in the order of 1–10 GΩ, popularly known as giga-seal. (C) After creating the giga-seal, more suction is applied to break into the cell to form the whole-cell mode without losing the giga-seal.

**Fig. 3:**
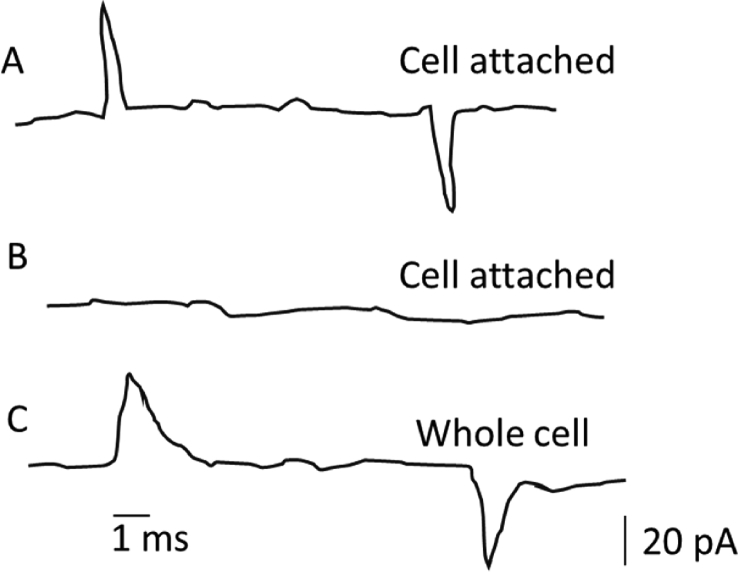
Electrical sequence of changes during the formation of a whole-cell mode. (A) When the giga-seal is formed, a transient current flows due to the intrinsic capacitance of the pipette. (B) The capacitance is adjusted by injecting an extra current through the pipette with the help of the amplifier. (C) After rupturing the cell membrane, when the whole-cell mode is achieved, a transient current is passed through the cell due to the capacitive nature of the cellular membrane.

The whole-cell patch-clamp method is briefly discussed below [18]. In a whole-cell patch-clamp, a known voltage or current is applied to a cell (if an external stimulus is required), and the responsive current or voltage is measured. The process of formation of a whole-cell patch-clamp configuration is described in Fig. 2. In this method, a glass microelectrode filled with an intracellular buffer is attached to the cell membrane to form a giga-seal with an electrical resistance on the order of gigaohms to prevent leakage of any current flowing between the cell membrane and the glass electrode. The electrical measurement process of the three-step–based whole-cell formation is described in Fig. 3 [18]. The complete electrical circuit mechanism of a whole-cell patch-clamp can be found elsewhere [15]. Patch-clamp is a versatile technique that has revolutionized medicine and physiology. Most studies utilizing this technique involve *in vitro* or *ex vivo* measurements.

#### Intracellular recording

In the intracellular recording, high resistance (20–40 MΩ) glass microelectrodes are filled with 1–3 M KCl solution and inserted into the cells to measure the membrane voltage. The total osmolarity of buffer in which cells are incubated is around 290–310 mOsm. The mouth size of the pipette is on the order of nanometers, which does not allow the flow of KCl solution from the pipette to the cells, and hence there is no osmotic shock. Intracellular recordings are helpful to measure the synaptic signals of cells with a high signal-to-noise ratio [19].

#### Microelectrode arrays

Microelectrode arrays allow the simultaneous detection of hundreds of sites in a neural network [20]. Microelectrode arrays are invasively implanted into the brain for *in vivo* experiments. One of the significant disadvantages of microelectrode-based arrays is that they require complicated surgery procedures, and the implanted arrays can lead to an unwanted immune response from the body.

#### Brain mesh electronics

Lieber and coworkers demonstrated a new technique called ‘Brain Mesh Electronics’ to record signals *in vivo* in mice brains with a resolution in the order of microvolts [17]. The injectable mesh electronics system can record signals at a single neuron level, perform recordings for at least eight months, and not elicit any chronic immune response [17,21]. However, this technique is highly invasive and can measure only up to a few hundred neurons at a time.

### Optical techniques

Optical-based molecular dyes offer several advantages to measure the membrane potential of excitable cells.

(1) Optical dyes can measure membrane potential simultaneously from multiple cell sites and multiple cells simultaneously [22]^.^
(2) The use of optical dyes does not require complicated surgical procedures.
(3) Optical dyes are not invasive.
(4) They provide a high spatial resolution in comparison to the microelectrode-based techniques.

Optical dyes are typically used through different imaging modalities such as (a) fluorescence [23], (b) second harmonic generation (SHG) [24], and (c) photoacoustic tomography [25,26]. The voltage sensing efficiency of an optical probe can be assessed by fractional signal intensity change, ΔF/F per 100 mV, where F is the baseline intensity generated by the probe without any electrical stimulation [27]. The sensitivity (ΔF/F) of a dye can be increased in several ways, such as by engineering the molecular structures of the probe, by increasing the power of the light source used to illuminate the dye, and by increasing the dye concentration. Increasing the probe concentration or the power of the light source often leads to toxicity and photodamage. By engineering the molecular structures of the probe, the sensitivity can be increased without any phototoxicity or photodamage. In the bid to measure voltage changes across the plasma membrane of the cell with high ΔF/F, numerous optical probes have been designed and reported. Apart from a high ΔF/F, an ideal voltage-sensitive probe shall give temporal resolution, should not show dark toxicity, shall not interfere with the cellular mechanisms, and shall not report any false positive or negative signals [28].

A majority of the optical probes are fluorescence-based, which are discussed below.

#### Fluorescent-based dyes

Fluorescent-based voltage-sensitive probes can be classified into (a) Indirect dyes and (b) Direct dyes. Indirect dyes measure the action potential by measuring the change of ion concentrations (e.g., Ca^2+^ or Na^+^) during an action potential [11,29]. Direct dyes are plasma membrane-bound dyes that respond directly to the membrane potential change by various mechanisms, such as electrochromism, Förster resonant energy transfer, and photo-induced electron transfer [[30], [31], [32]]. Below, I have briefly described the indirect and direct dyes.

##### Indirect dyes

**Fig. 4:**
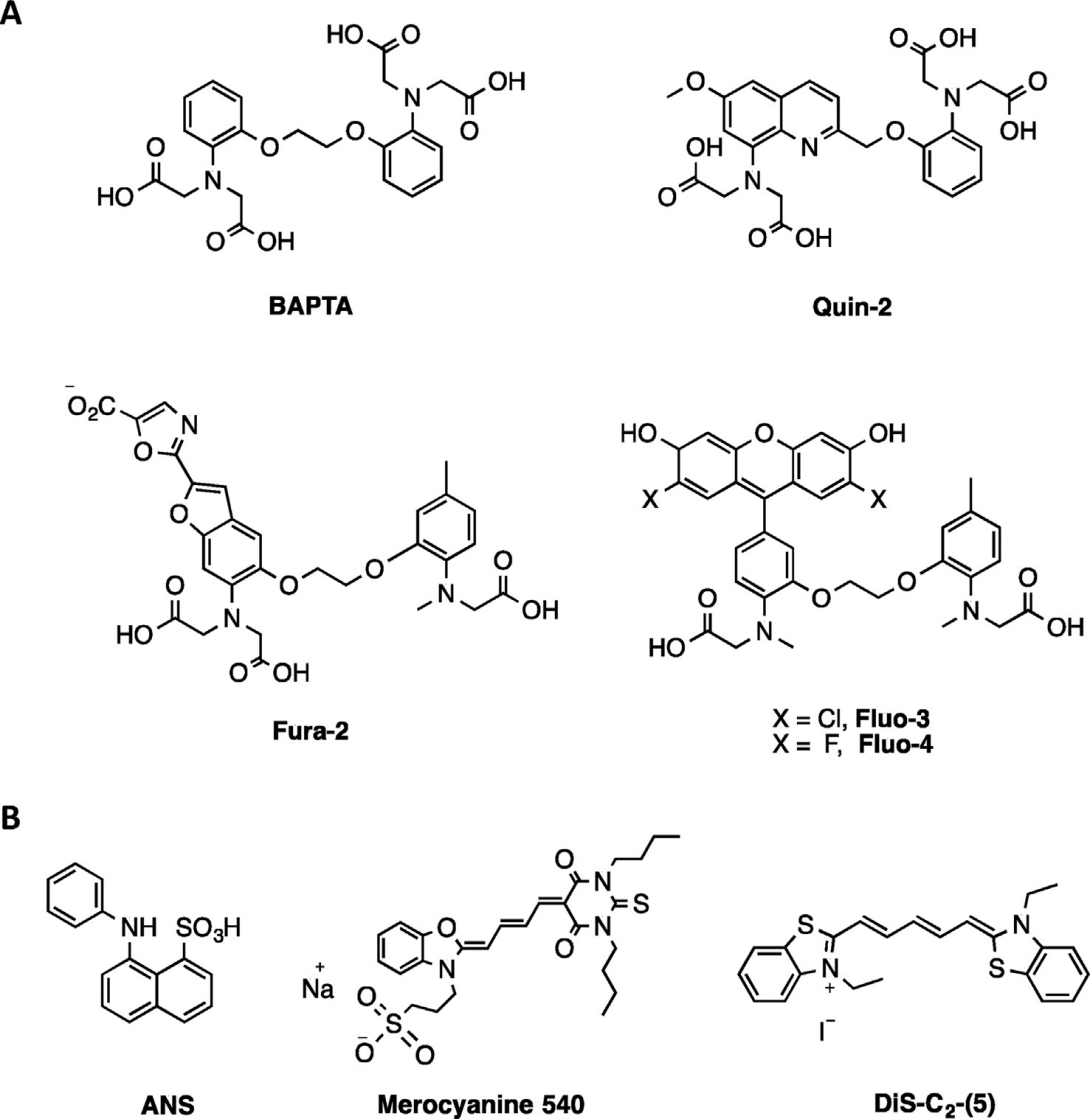
Chemical structures of a calcium chelator, BAPTA, and the calcium-sensitive fluorescent probes, Quin-2, Fura-2, Fluo-3, and Fluo-4. (B) Molecular structures of 8-anilinonaphthalene-1-sulfonic acid (ANS), Merocyanine-540, and DiS-C_2_-(5) dyes.

Calcium-sensitive fluorescent dyes have been the most successful indirect dyes used to measure the action potential of neurons. Calcium-sensitive probes respond to the change in calcium concentration during an action potential. Tsien and coworkers developed one of the first calcium-sensitive dyes, such as Quin-2, Fura-2, Fluo-3, and Fluo-4, which are a combination of a fluorescent chromophore and a calcium-binding chelator, BAPTA [Fig. 4A] [11,23,33]. These dyes generated ΔF/F up to a value of 50% per 100 mV but gave a poor temporal resolution [34,35]. Since these dyes measure the change in calcium concentrations resulting from an action potential, there is always a delay. The temporal resolutions of the calcium-based dyes are dependent on the rate of diffusion of calcium ions through the cell body. Action potentials in neurons occur in a timescale of 2 ms, but these dyes respond in a timescale of seconds. Moreover, small molecule-based calcium dyes could not be easily used for *in vivo* experiments due to poor neuronal delivery and toxicity.

Tsien and coworkers designed a new generation of protein-based calcium dyes known as genetically encoded calcium indicators (GECIs) to overcome the issues of low temporal resolution and inefficient *in vivo* delivery of the organic dyes [36]. These dyes give better temporal resolutions and are compatible with different types of cells both *in vitro* and *in vivo*. Numerous literature is available on their working mechanisms and types, which can be found elsewhere [37,38].

Despite significant developments, calcium-based dyes have several disadvantages. In several cases, the calcium-binding chelators perturb calcium homeostasis, thus affecting the cellular electrical activity [34,39]. Due to calcium saturation, many calcium-based dyes cannot retain their efficiency over time. The GECIs have been found to exhibit better temporal resolution than small molecule-based calcium dyes, but often they do not reflect reliable calcium signals associated with a single action potential [40]. Moreover, GECIs cannot measure subthreshold repolarization and depolarization events and are restricted to measuring only action potential spikes [41].

##### Direct dyes

The plasma membrane-bound amphiphilic direct dyes were designed to sense the membrane potential change directly. The initial class of direct dyes such as 8-anilinonaphthalene-1-sulfonic acid (ANS), Merocyanine 540, and DiS-C_2_-(5) [Fig. 4B] worked by undergoing macromolecular changes such as rotation, dimerization, and reorientation in the cell membrane due to an action potential leading to a change in their fluorescence signals. These dyes were reported to have either poor sensitivity (ΔF/F ≈ 0.001–0.1%) or poor temporal resolution (seconds to minutes) [[42], [43], [44]]. The next generation of direct dyes with better sensitivities and temporal resolutions dependent on molecular electronic changes are divided into three types: (i) Electrochromic dyes, (ii) Förster resonance energy transfer (FRET)-based dyes, and (iii) Photo-induced electron transfer (PeT)-based dyes.

###### Electrochromic dyes

Electrochromic dyes are based on the molecular Stark effect to generate a direct electro-optic response to an applied electric field [45,46]. In electrochromism, the absorption and emission spectra of a chromophore change (i.e., change in electronic energy levels) when placed in a local electric field.

**Fig. 5:**
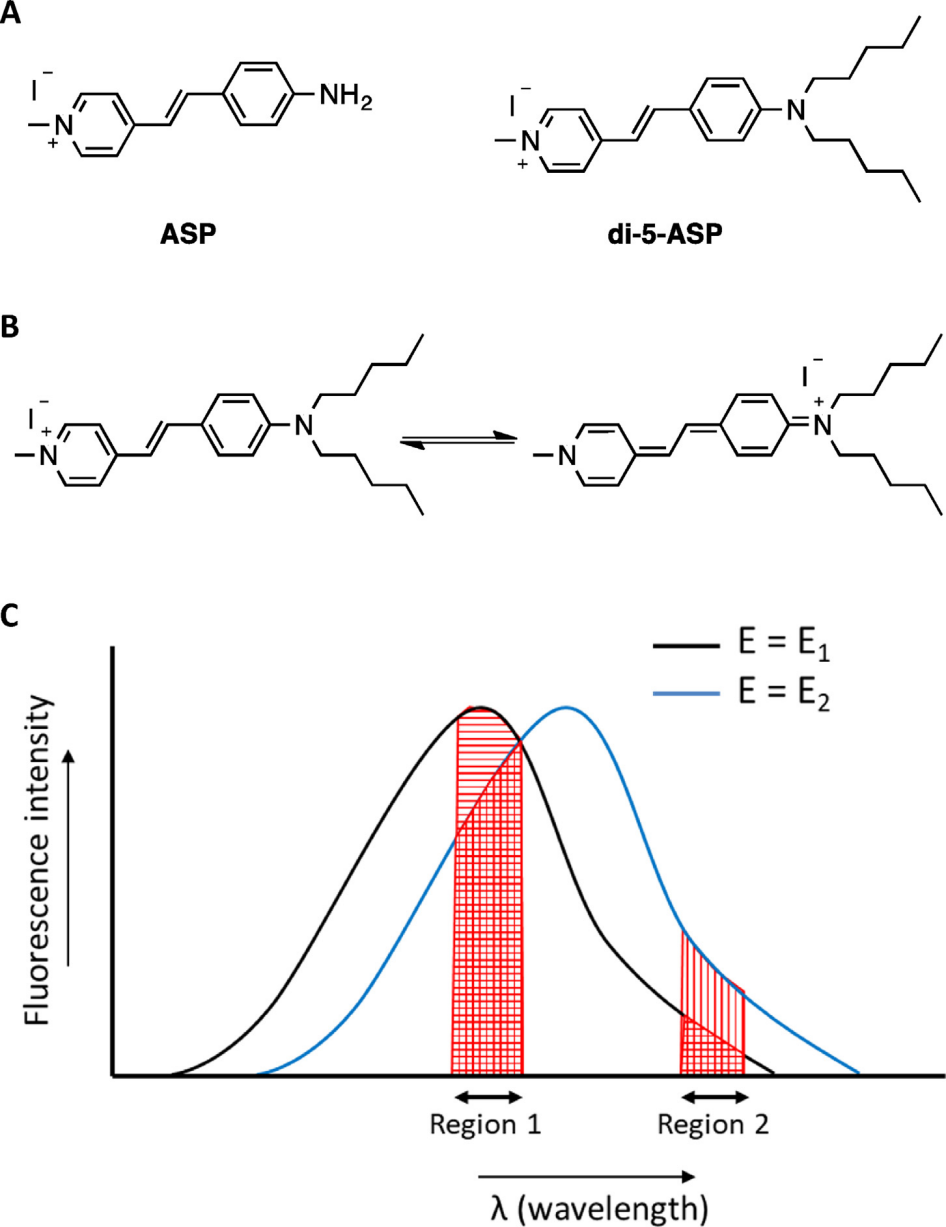
(A) Molecular structures of ASP and di-5-ASP. (B) Resonance structures of di-5-ASP showing distribution of charge in the ground and first electronic excited states. (C) Pseudo emission spectra to demonstrate electrochromism. Due to the shift of the emission spectrum towards the right side at the electric field, E = E_2_ from E_1_, higher voltage-sensitivity (change in the spectrum) can be recorded by collecting photons from the tail (region 2) than in the middle (region 1).

For a chromophore to respond to the changing electric field, it must follow two primary requirements: (a) The chromophore dipole must be rigidly and anisotropically oriented with respect to the electric field [47], and (b) the chromophore should exhibit a significant shift in the electron density distribution upon excitation by light. Loew and coworkers designed an amphiphilic donor–acceptor–based dye, di-5-ASP, based on the structure of 4-aminostyryl-1-methylpyridinium iodide [ASP, Fig. 5A] [48,49]. The alkyl chains of di-5-ASP [Fig. 5A] fit the hydrophobic core of the membrane while the charged part at the other end of the dye strongly interacts with the hydrophilic part of the membrane, thus satisfying the first criterion of rigid and anisotropic orientation. In the ground state, the electron density is localized in the aniline ring, while in the first excited state, it is localized in the pyridinium ring [Fig. 5B], thus exhibiting a significant shift in the electron density on photoexcitation, which is the second criterion for an electrochromic chromophore. This dye was reported ΔF/F = 5% per 100 mV in a hemispherical lipid bilayer [50]. Since electrochromism is based on the shift in the absorption or emission spectrum, the voltage sensitivities of electrochromic dyes depend on the wavelengths of the spectra at which the microscope detector collects the photons. An electrochromic dye gives higher voltage sensitivity at the tail region [region 2, Fig. 5C] than at the middle of the spectrum [e.g., region 1, Fig. 5C].

**Fig. 6:**
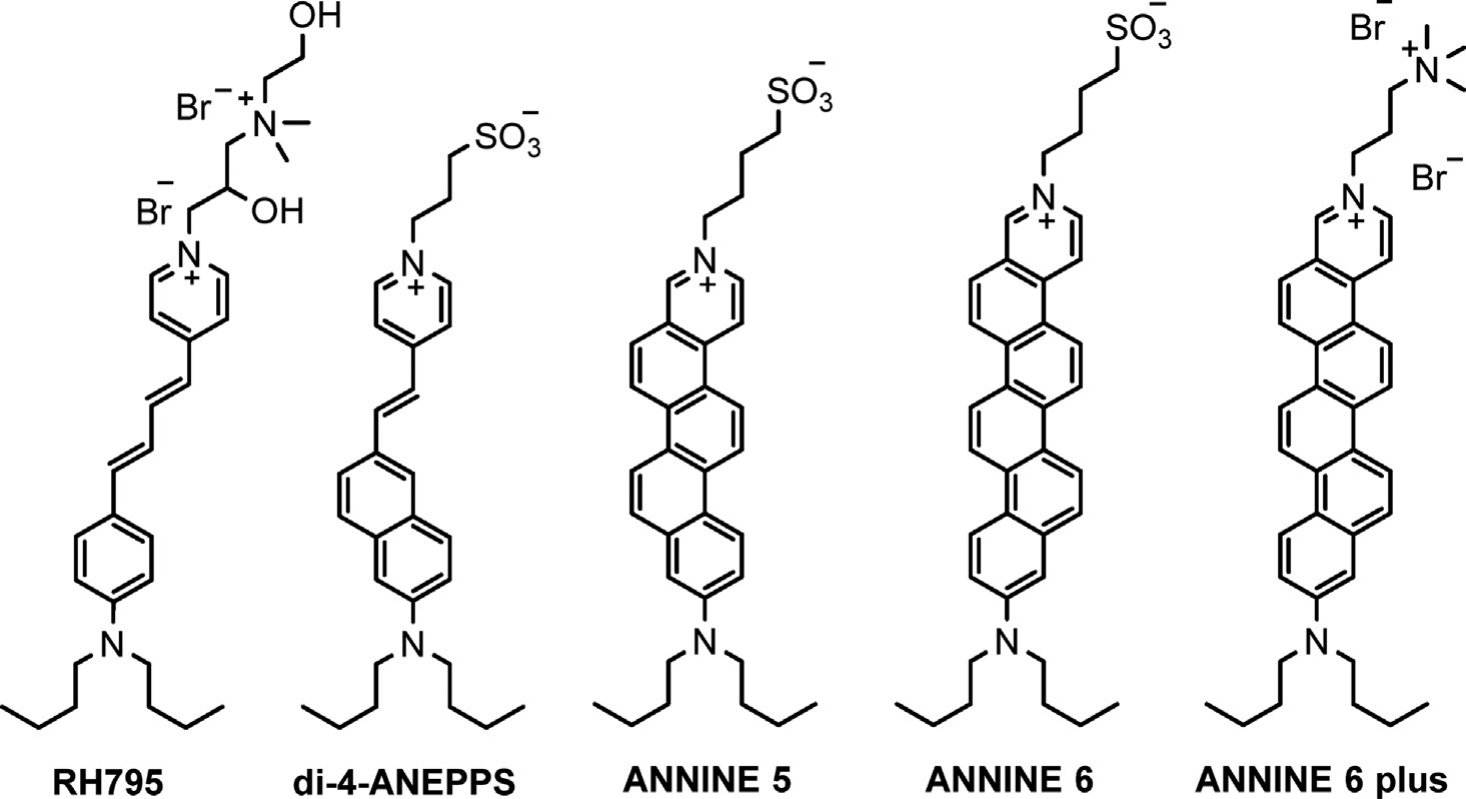
Molecular structures of RH795, di-4-ANEPPS, ANNINE-5, ANNINE-6, and ANNINE-6plus.

The dipole moment of a dye becomes stronger when the length of electronic conjugation between its electron donor and acceptor moieties increases [51,52]. Hence, dyes with longer electronic conjugation should have greater electrochromic shifts. Additionally, in a plasma membrane, the maximum change in the transmembrane potential occurs at the middle of the bilayer across the fully dehydrated region [28,52]. Since the typical thickness of the lipid bilayer in a plasma membrane is 4–5 nm, by increasing the overall size of the dye molecule (size of di-5-ASP ≈ 1.7 nm), a greater electrochromic change can be achieved. Hildesheim and coworkers developed a new class of RH-based (Rina Hildesheim) dyes such as RH795 [Fig. 6] by extending the conjugation of ASP dye by adding extra ethene linkers and modifying the polar headgroups [53,54]. Loew and coworkers designed a new class of naphthalene-based amphiphilic ANEP (amino naphthenyl-pyridinium) dyes [e.g. Di-4-ANEPPS, Fig. 6] with extended conjugations and studied their voltage sensitivity in artificial lipid bilayers and live cells [55,56]. One of the major issues with the styryl-based dyes is that they undergo cis–trans photoisomerism, which contributes to shifts in the absorption and emission spectra, making membrane potential measurements not completely reliable [52,57]. Fromherz and coworkers synthesized several rigid dyes, ANNINE-5, ANNINE-6, ANNINE-6plus [Fig. 6], that do not undergo photoisomerism [52,58]. The ANNINE-based dyes gave very high voltage sensitivity with ΔF/F as high as 50% per 100 mV [57,58]. Although electrochromic dyes have emerged as powerful tools to measure voltage changes in cellular plasma membranes, they have a general disadvantage. The maximum voltage sensitivity is observed only at the tails of the emission spectra, where the number of emitted photons is low, leading to a poor signal-to-noise ratio. Moreover, electrochromic dyes are polarity-sensitive and thus exhibit solvatochromism, which perturbs the pure electrochromic response [59]. Lipids in the plasma membrane undergo polarity changes due to biological events, because of which they become tightly packed or loosely packed. When an electrochromic dye is used for membrane potential measurements, the contribution of changes in lipid order to the fluorescence emission of the dye cannot be segregated [59].

###### Förster resonance energy transfer (FRET)-based dyes

**Fig. 7:**
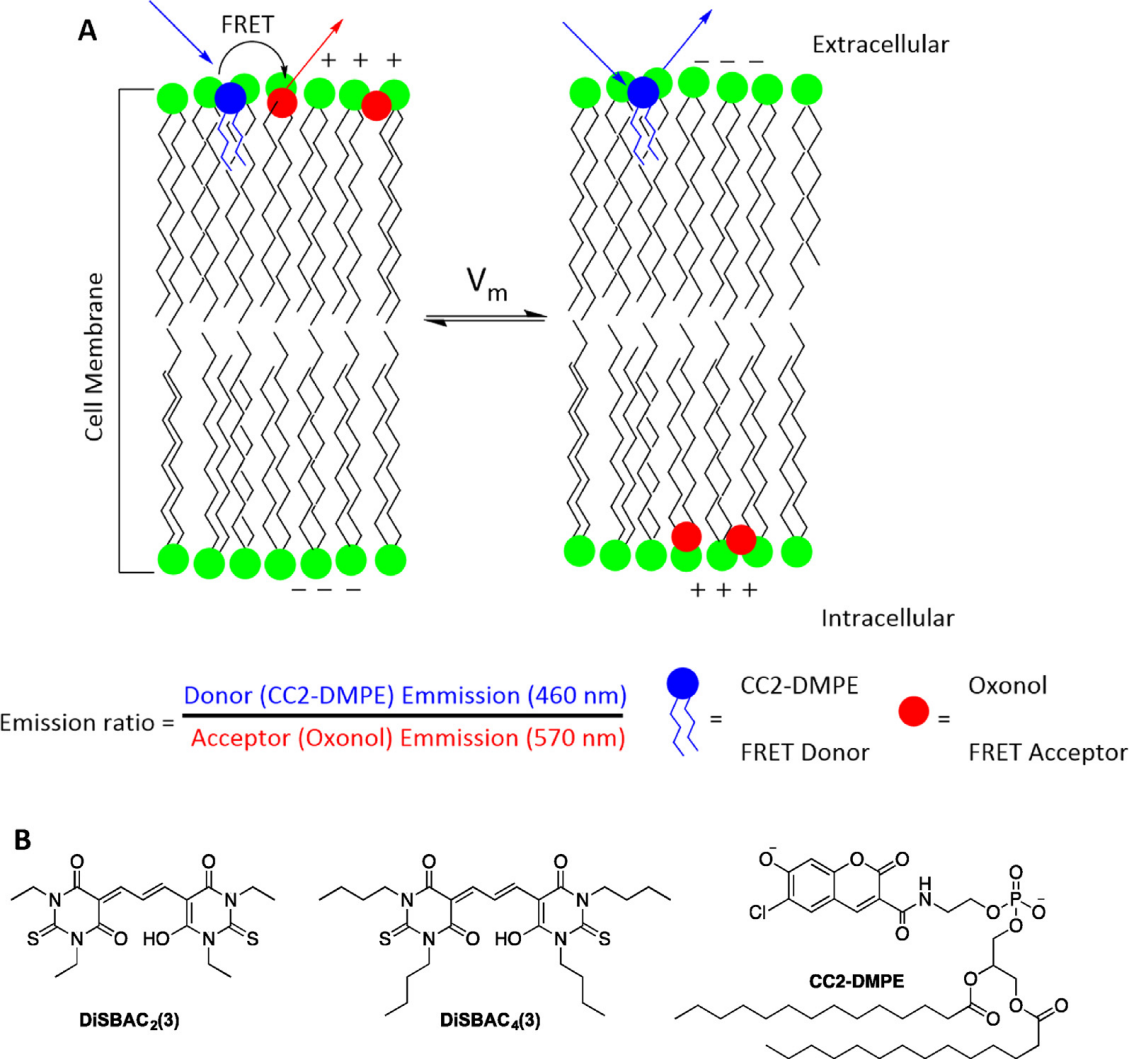
(A) Depiction of the FRET mechanism. In resting membrane potential (intracellular is negative), both molecules of the FRET pair bind to the extracellular leaflet of the cell membrane leading to efficient FRET. After depolarization (intracellular is positive), the anionic mobile acceptor (oxonol) shifts to the intracellular leaflet of the cell membrane while the donor remains bound to the outer surface, thus quenching the FRET. The fluorescence intensity ratio of the donor to acceptor molecules denotes the changes in membrane potential. (B) Molecular structures of oxonol FRET-based acceptor dyes DiSBAC_2_(3) and DiSBAC_4_(3), and the donor dye, CC2-DMPE.

Tsien and coworkers first designed FRET-based voltage-sensitive dyes [60]. FRET-based dyes use a pair of chromophores where one chromophore is a membrane bound energy-donor while the other chromophore is a membrane bound mobile voltage-sensitive energy-acceptor. When the cell is at resting membrane potential, both the donor and acceptor chromophores are bound to the outer leaflet of the plasma membrane. This is because the amphiphilic donor chromophore is designed to bind permanently to the extracellular leaflet of the dye while the anionic acceptor chromophore binds to the extracellular leaflet due to electrostatic interaction [Fig. 7A]. At this state, Förster resonant energy transfer takes place from the donor chromophore to the acceptor chromophore, resulting in the quenching of fluorescence emission of the donor chromophore and enhancement of fluorescence emission of the acceptor chromophore. As the membrane potential changes, the cell becomes depolarized, because of which the anionic mobile acceptor travels towards the intracellular leaflet of the plasma membrane due to electrostatic interaction. As the mobile acceptor travels, quenching of FRET takes place, resulting in a change in the fluorescence. The ratio of change in fluorescence emissions of the donor and acceptor chromophore denotes the change in membrane potential [Fig. 7A]. For FRET to take place, the acceptor chromophore must be in the vicinity of the donor chromophore because the efficiency of FRET probes is proportional to $\frac{R_{0}}{r^{6}}$, where r is the distance between the two chromophores and $R_{0}$ is Förster radius (distance between the donor and the acceptor at which 50% of the energy is transferred) [61]. In conventional donor–acceptor pairs of FRET-based dyes, the $R_{0}$ value lies in the range of 2–8 nm, which is similar to the thickness of cellular plasma membrane, and hence it is possible to use these dyes to measure the membrane potential of cells [62]. Anionic oxonol-based dyes such as DiSBAC_2_(3) and DiSBAC_4_(3) [Fig. 7B] are used as FRET acceptor chromophores in combination with coumarin phospholipid, CC2-DMPE as the donor chromophore [Fig. 7B] [63]. Pair of CC2-DMPE and DiSBAC_2_(3) as a FRET-based system has been shown to give voltage sensitivity, ΔF/F up to 300% in live cells [64].

FRET-based dyes have some disadvantages. The donor and acceptor chromophores of FRET-based dyes require precise stoichiometry, which is difficult to achieve in most cases due to the low aqueous solubility of the hydrophobic mobile acceptors. For example, DiSBAC_4_(3) requires Pluronic F-127 surfactant or DMSO for staining. Other chromophores such as DiSBAC_2_(3) do not require Pluronic F-127 or DMSO as they are more polar, but they need to be loaded in high concentrations up to 20 mM. Such a high concentration of chromophores can affect the resting potential of the cells, as they tend to increase cell capacitance [65]. Moreover, the temporal resolution of the FRET mechanism is dependent on the translocation of the mobile acceptor inside the cell membrane, and hence it is not a purely electro-optic response.

###### Dyes based on photo-induced electron transfer (PeT) through molecular wires [66]

**Fig. 8:**
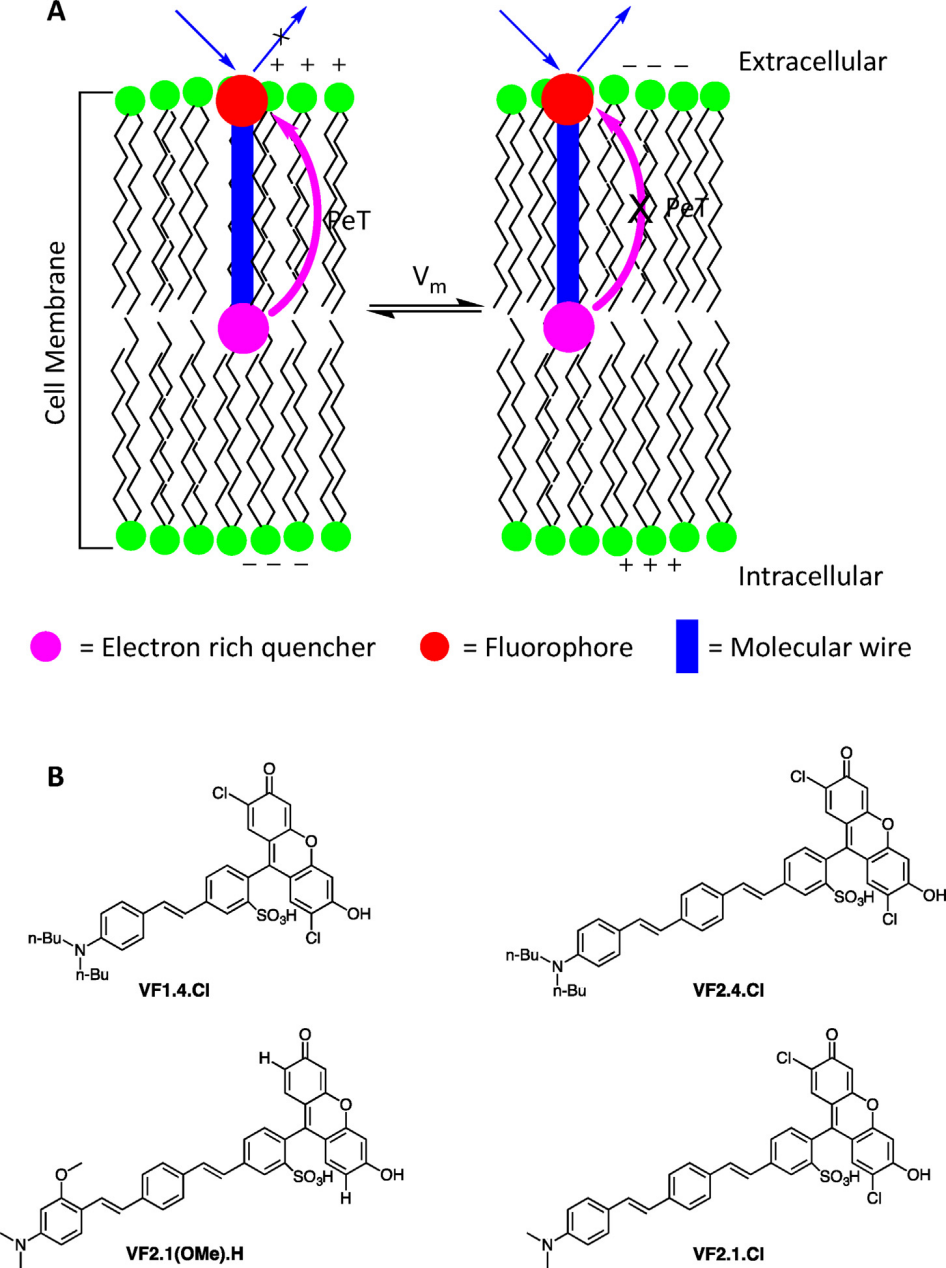
(A) Depiction of photo-induced electron transfer (PeT) mechanism. When the cell is at resting potential, PeT takes place from the electron-rich quencher to the fluorophore *via* the molecular wire. As the cell depolarizes, the PeT becomes less prominent, and hence the fluorophore emits light. The change in fluorescence intensity represents the change in membrane potential. (B) Molecular structures of PeT-based dyes.

Tsien and coworkers first designed and demonstrated the use of PeT-based dyes to measure membrane potential in live cells [67]. The PeT-based voltage-sensitive dyes contain a fluorophore, an electron-rich donor moiety known as the fluorescence quencher, and a molecular wire connecting the electron-rich quencher to the fluorophore. In PeT, when the molecule is in an electronically excited state, electron transfer takes place from the electron-rich quencher to the fluorophore *via* the molecular wire to quench the fluorescence of the fluorophore [Fig. 8A]. The amphiphilic dye is designed such that the fluorophore binds to the extracellular leaflet of the plasma membrane, and the electron-rich quencher moiety binds towards the intracellular leaflet of the plasma membrane [Fig. 8A]. When the cell is at resting potential, the PeT transfer efficiency is highest. As the voltage across the cell membrane changes and the plasma membrane depolarizes, the efficiency of the PeT is affected, and hence, there is a change in fluorescence intensity of the fluorophore. The change in the fluorescence denotes the change in membrane potential. An advantage of PeT-based dyes over FRET-based dyes is that the temporal resolution is dependent on the intramolecular electron transfer, which takes place in a time range of femtoseconds to picoseconds, and hence, the dyes could be used to resolve fast action potential spikes in neurons [67].

The initial PeT-based dyes incorporated dichlorosulfofluorescein as the membrane bound fluorophore, *N,N*-dimethyl- or *N,N*-dibutylaniline as the electron-rich quencher moiety, and *p-*phenylenevinylene (PPV) as the molecular wire to synthesize three dyes, VF1.4.Cl, VF2.1.Cl and VF2.4.Cl [Fig. 8B] [67]. When tested in leech neurons, these dyes did not show any capacitive loading, making them favorable over FRET-based dyes. The dyes exhibited fast kinetics and reported voltage sensitivity ΔF/F of approximately 20–27% per 100 mV. Tsien and coworkers improved the design to synthesize VF2.1(OMe).H [Fig. 8B] with voltage sensitivity (ΔF/F) as high as 48% per 100 mV, which is twice the sensitivity of the previous PeT-based dyes [30]. Apart from these dyes, several PeT-based dyes have been synthesized by Miller and coworkers with increased voltage sensitivity, plasma membrane binding efficiency, and photostability [68].

Although PeT-based voltage-sensitive dyes have given promising results with good voltage sensitivity and temporal resolution (in the order of milliseconds), there are a few challenges that remain associated with them. For an efficient PeT, all the dye molecules must be aligned in a similar orientation in the cellular membranes; however, such an orientation does not always occur, leading to decreased efficiency. Moreover, the PeT efficiency decreases exponentially with the increase in the length of molecular wires [69]. A PeT-based dye should span across the whole plasma membrane to efficiently sense the voltage change across the plasma membrane. However, the longer the dye, the lower the PeT efficiency, and thus a compromise must be made between efficient PeT and efficient voltage sensing. Further, the existing PeT-based dyes are styryl-based and may undergo photoisomerism leading to an unwanted change in PeT efficiency.

As described above through three different mechanisms, electrochromism, FRET, and PeT, the small molecule-based voltage-sensitive dyes offer good voltage sensitivity and temporal resolution and are easy to deliver in cells; however, there are certain limitations associated with each of them. For example, none of these dyes can be used for long-term measurements for days to months because they tend to clear away from the cells. *In vivo* delivery of these dyes remains an issue and requires invasive intracranial surgeries. As briefly discussed below, genetically encoded protein-based dyes have been designed to overcome these challenges.

##### Genetically encoded voltage indicators (GEVIs)

Isacoff and coworkers reported the first genetically encoded voltage sensor in 1997 [12]; however, it was only after 2005 that the research field of GEVIs gained pace when Okamura and coworkers discovered a single protein-based voltage-sensitive phosphatase [70]. Since then, different types of GEVIs have been demonstrated that utilize voltage-sensing domains, electrochromic rhodopsins [71,72], and dual opsin-based FRET mechanisms [31,73]. A few challenges remain to be addressed in the field of GEVIs. GEVIs can be targeted to specific cell types, which is not a trivial task in the case of small molecules [74]. Unlike small molecule-based voltage indicators, GEVIs do not require invasive craniotomy to be delivered *in vivo* and can be used for long-term (days to months) experiments [75]. GEVIs have also been shown to record spikes in neurons of awake mice [73]. Sometimes these indicators may cross the plasma membrane and accumulate inside the cells [76]. The expression of GEVIs in neurons is not trivial and requires advanced experimental skills. Nonetheless, newer studies are being reported with better GEVIs, which are pushing the frontiers of neurobiology [[77], [78], [79]]. A more detailed review of GEVIs can be found elsewhere [41,78].

#### Second harmonic generation (SHG)

Second-harmonic generation (SHG) is a nonlinear optical process that occurs when a medium of high quadratic hyperpolarizability encounters an intense, focused, and coherent laser light to generate light of exactly twice the frequency [80]. SHG occurs only at the interfaces populated with an ensemble of non-centrosymmetric molecules with ordered first-order hyperpolarizabilities [80]. Since SHG is not observed in isotropic media [81], it is useful for measuring the plasma membrane potential of cells without any background signals [82,83]. SHG is a scattering technique and does not involve a population of excited states because of which there is no phototoxicity or photobleaching. It must be noted that since SHG is a scattering technique, the SHG light is scattered only in the forward direction, i.e., towards the direction at which the incident light travels. Unlike fluorescence, SHG light is weak in the reflected direction, and hence it cannot be used for brain-related *in vivo* experiments, which is a major disadvantage of SHG-based imaging. Physically, SHG is a hyper-Rayleigh scattering technique, so SHG of any molecule depends on its first-order hyperpolarizability [84]. The SHG-based voltage sensitivity of a molecule depends on its second-order hyperpolarizability, and so for any molecule to be able to give a reliable change of SHG signals upon a change in the electric field, it must have high first-order and second-order hyperpolarizabilities [80,85].

##### SHG-based voltage-sensitive dyes

**Fig. 9:**
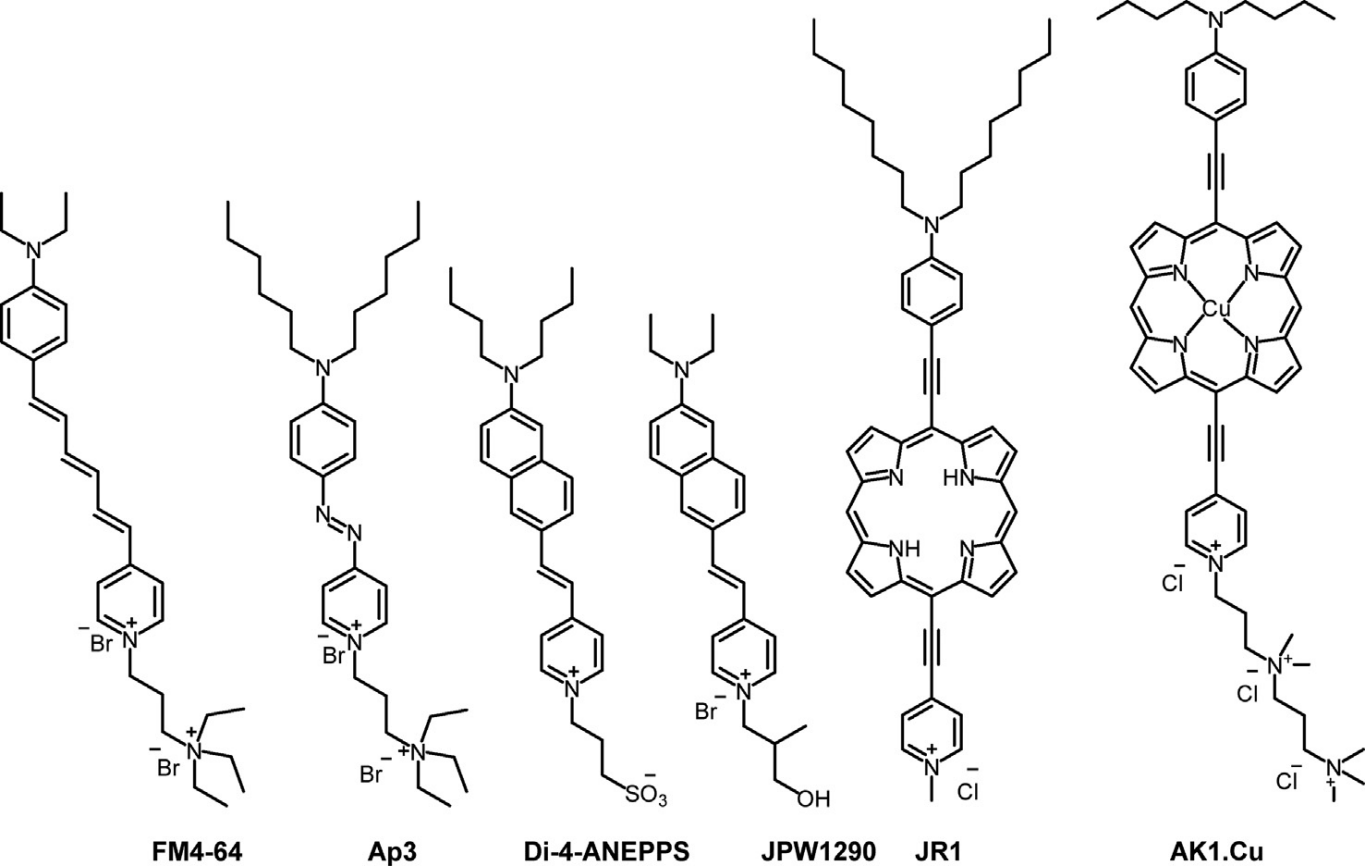
Chemical structures of SHG-based voltage-sensitive dyes, FM4-64, Ap3, di-4-ANEPPS, JPW1290, JR1, and AK1.Cu.

Loew and coworkers first reported that SHG could be used to measure membrane potential using styryl-based dyes, such as di-4-ANEPPS and JPW1290 [Fig. 9] in hemispherical lipid bilayers and photoreceptor cells of white-eyed Musca flies [83,86]. Since the work of Loew and coworkers, several styryl-based dyes have been shown to record membrane potential in lipid bilayers and live cells by SHG [24,85,87]. Dombeck et al. tested the voltage sensitivity FM4-64 [Fig. 9] in both cultured neuronal cells and neurons in rat brain slices using the patch-clamp technique [24,88]. They compared both fluorescence and SHG –based voltage sensitivities of FM4-64 with ANNINE-6. As expected, the fluorescence-based voltage sensitivity of ANNINE-6 was 40 times higher than that of FM4-64; however, the SHG-based voltage sensitivities of both the dyes were similar. In neurons of mouse brain slices, ANNINE-6 [Fig. 6] did not localize in the plasma membrane. It must be noted that FM4-64 is a dicationic dye while ANNINE-6 is a sulfonate-based zwitterionic dye. Khadria et al. described that dicationic dyes are more effective in plasma membrane localization than sulfonate-based dyes [89]. Nuriya et al. developed SHG only photostable azo-based dye, Ap3 [Fig. 9], with negligible fluorescence [87]. Anderson and coworkers synthesized a new class of porphyrin-based voltage-sensitive dye, JR1 [Fig. 9], and SHG only AK1.Cu [Fig. 9], which has higher plasma membrane binding efficiency [85,89,90].

#### Challenges with fluorescence and SHG-based techniques

Fluorescent-based voltage-sensitive dyes have revolutionized the field of neurobiology because of their ability to test membrane potential from multiple neurons simultaneously. However, there are issues associated with the principle of fluorescence microscopy, which has posed significant challenges in bioimaging.

##### Deep imaging

Focused light utilized in both the fluorescence and SHG microscopies cannot penetrate deep tissues beyond 800–1000 μm because the light gets absorbed or scattered by the tissues and blood [91]. Due to light penetration issues, it has not been possible to image thick biological tissues or neurons located deep in mice brains.

##### Phototoxicity and photobleaching

Fluorescence works by the principle of photoexcitation of a fluorophore from the electronic ground state to an excited state resulting in radiative decay (fluorescence) back to the ground state [92]. Due to the involvement of excited states, molecular oxygen present in the medium can be transformed to toxic reactive oxygen species that are harmful to both the biological species (phototoxicity) and the molecular dyes (photobleaching) [93,94]. Photobleaching is a significant concern for voltage-sensitive dyes, leading to new types of photostable dyes or the usage of oxygen scavengers [55,88,95]. Only a few fluorescent voltage-sensitive dyes have been reported, which are photostable for up to 15–20 min of continuous imaging, but long-term imaging (hours to days) of these dyes is yet to be demonstrated [77,95]. Although SHG does not involve the formation of excited states, only two dyes have been reported until now, which gives only SHG signal without any collateral fluorescence [87,89].

##### Photodamage of the biological sample

The absorption maxima of a majority of the fluorophores lie in the blue and green regions of the visible light spectrum. The use of blue and green light can lead to photodamage of cells; for example, blue light is has been linked to mitochondrial DNA damage [96].

##### Background signal

During fluorescence imaging, unwanted background fluorescence causes a decrease in the measured voltage sensitivity [90]. For potential membrane experiments, the dyes must localize at the plasma membrane of the cells; however, it has been a challenge to stop the dyes from being internalized into the cells due to cellular trafficking. Since most of the conventional dyes are gradually taken up by the cells, they result in background fluorescence which interferes with the fluorescence signal of interest.

## Conclusions

This review summarizes the working principles, advantages, and disadvantages of several techniques employed to measure the membrane potential of neurons. As discussed above, all the techniques have their own merits, but none are immune to disadvantages. An ideal technique should be able to measure the membrane potential of a large number of neurons at a high spatiotemporal resolution for a long time in deep tissues *in vivo* without losing efficiency, without perturbing the neuronal dynamics, and without generating any false positives or negatives. Although electrode-based techniques remain one of the preferred techniques for neurobiology, fluorescence-based methods have come a long way and are increasingly used. With the advent of optogenetics, an increasing number of neuroscientists are using fluorescence-based techniques. SHG has its advantages, but because it cannot be used *in vivo* in mice brains, it is unlikely to be considered one of the mainstream membrane potential sensing techniques.

Almost all the optical indicators are used to measure action/membrane potentials from cultured neurons *in vitro*, neurons in brain slices *ex vivo*, or mouse cortex neurons *in vivo* using one-photon or two-photon microscopy techniques. One of the most significant disadvantages of these microscopy techniques is that they cannot image deep tissues as the ballistic regime of light is limited to 1 mm in biological tissues [97]. Fiber optic technology is used to image deeper tissues, but it requires invasive surgical methods, which can perturb the inherent properties of neurons [98]. Non-invasive techniques such as photoacoustic imaging has recently gained traction to image neuronal activity from the mouse brain and live cells [99,100]. The use of photoacoustic imaging to sense neuronal activity is in its developmental stage. Only the light of wavelengths longer than 650 nm can penetrate deeper in biological tissues, but not many voltage-sensitive dyes exist that absorb light longer than 650 nm to be used as effective photoacoustic-based contrast agents.

With the increasing and diversified research on tools to map the whole brain activity, it is just a matter of time before we can completely understand the powerhouse of the human body that we call the brain.

## Funding

A.K. acknowledges the Clarendon Scholarships, Hilla Ginwala Scholarships, Radhakrishnan Memorial Fund, and St Catherine's College Overseas Graduate Scholarships at the 10.13039/100011348University of Oxford. This work was also supported by 10.13039/501100000266EPSRC (grants EP/G03706X/1 and EP/G037280/1).

## Conflicts of interest

The author declares no conflicts of interest.

## References

[1] von Bartheld C.S., Bahney J., Herculano-Houzel S. (2016). The search for true numbers of neurons and glial cells in the human brain: a review of 150 years of cell counting. J Comp Neurol.

[2] Harrison P.J., Freemantle N., Geddes J.R. (2003). Meta-analysis of brain weight in schizophrenia. Schizophr Res.

[3] Cajal SR.y. (1888). Estructura de los centros nerviosos de las aves. Rev Trim Hist Norm Pat.

[4] Sherrington C.S. (1906). Observations on the scratch-reflex in the spinal dog. J Physiol.

[5] Matteucci C. (1842). Deuxième mémoire sur le courant électrique propre de la grénouille et sur celui des animaux à sang chaud. Ann Chim Phys III.

[6] Bean B.P. (2007). The action potential in mammalian central neurons. Nat Rev Neurosci.

[7] Neher E., Sakmann B. (1976). Single-channel currents recorded from membrane of denervated frog muscle fibres. Nature.

[8] Galvani L. (1791). De viribus electricitatis in motu muscularis commentarius. Bononiensi Sci Artium Inst Atque Acad Comment.

[9] Bernstein J. (1868). Ueber den zeitlichen Verlauf der negativen Schwankung des Nervenstroms. Pflugers Arch J Physiol.

[10] Bernstein J. (1902). Untersuchungen zur Thermodynamik der bioelek- trischen Ströme. Pflugers Arch J Physiol.

[11] Tsien R.Y. (1980). New calcium indicators and buffers with high selectivity against magnesium and protons: design, synthesis, and properties of prototype structures. Biochemistry.

[12] Siegel M.S., Isacoff E.Y. (1997). A genetically encoded optical probe of membrane voltage. Neuron.

[13] Hodgkin A.L. (1976). Chance and design in electrophysiology: an informal account of certain experiments on nerve carried out between 1934 and 1952. J Physiol.

[14] Martin J.K. (1963). The 1963 Nobel prize in Physiology or medicine. Can Med Assoc J.

[15] Halliwell J.V., Whitaker M.J., Ogden D. (1994).

[16] Churchland M.M., Yu B.M., Sahani M., Shenoy K.V. (2007). Techniques for extracting single-trial activity patterns from large-scale neural recordings. Curr Opin Neurobiol.

[17] Fu T.M., Hong G., Zhou T., Schuhmann T.G., Viveros R.D., Lieber C.M. (2016). Stable long-term chronic brain mapping at the single-neuron level. Nat Methods.

[18] Ogden D., Stanfield P. (1994).

[19] Li W.C., Soffe S.R., Roberts A. (2004). A direct comparison of whole cell patch and sharp electrodes by simultaneous recording from single spinal neurons in frog tadpoles. J Neurophysiol.

[20] Meister M., Pine J., Baylor D.A. (1994). Multi-neuronal signals from the retina: acquisition and analysis. J Neurosci Methods.

[21] Zhou T., Hong G., Fu T.M., Yang X., Schuhmann T.G., Viveros R.D. (2017). Syringe-injectable mesh electronics integrate seamlessly with minimal chronic immune response in the brain. Proc Natl Acad Sci U S A.

[22] Zochowski M., Wachowiak M., Falk C.X., Cohen L.B., Lam Y.W., Antic S. (2000). Imaging membrane potential with voltage-sensitive dyes. Biol Bull.

[23] Minta A., Kao J.P., Tsien R.Y. (1989). Fluorescent indicators for cytosolic calcium based on rhodamine and fluorescein chromophores. J Biol Chem.

[24] Dombeck D.A., Sacconi L., Blanchard-Desce M., Webb W.W. (2005). Optical recording of fast neuronal membrane potential transients in acute mammalian brain slices by second-harmonic generation microscopy. J Neurophysiol.

[25] Khadria A., Paavola C.D., Zhang Y., Davis S.P.X., Grealish P., Maslov K. (2022). Long-duration and non-invasive photoacoustic imaging of multiple anatomical structures in a live mouse using a single contrast agent. bioRxiv.

[26] Khadria A., Paavola C.D., Maslov K., Valenzuela F.A., Sperry A.E., Cox A.L. (2022). Photoacoustic imaging reveals mechanisms of rapid-acting insulin formulations dynamics at the injection site. Mol Metabol.

[27] Cohen L.B., Canepari M., Zecevic D. (2010). Membr. Potential imaging Nerv. Syst. Methods Appl..

[28] Peterka D.S., Takahashi H., Yuste R. (2011). Imaging voltage in neurons. Neuron.

[29] Minta A., Tsien R.Y. (1989). Fluorescent indicators for cytosolic sodium. J Biol Chem.

[30] Woodford C.R., Frady E.P., Smith R.S., Morey B., Canzi G., Palida S.F. (2015). Improved PeT molecules for optically sensing voltage in neurons. J Am Chem Soc.

[31] Gong Y., Wagner M.J., Zhong Li J., Schnitzer M.J. (2014). Imaging neural spiking in brain tissue using FRET-opsin protein voltage sensors. Nat Commun.

[32] Loew L.M. (1982). Design and characterization of electrochromic membrane probes. J Biochem Biophys Methods.

[33] Tsien R.Y., Pozzan T., Rink T.J. (1982). Calcium homeostasis in intact lymphocytes: cytoplasmic free calcium monitored with a new, intracellularly trapped fluorescent indicator. J Cell Biol.

[34] Smetters D., Majewska A., Yuste R. (1999). Detecting action potentials in neuronal populations with calcium imaging. Methods.

[35] Yuste R., Denk W. (1995). Dendritic spines as basic functional units of neuronal integration. Nature.

[36] Miyawaki A., Llopis J., Heim R., McCaffery J.M., Adams J.A., Ikura M. (1997). Fluorescent indicators for Ca2+ based on green fluorescent proteins and calmodulin. Nature.

[37] Chen T.W., Wardill T.J., Sun Y., Pulver S.R., Renninger S.L., Baohan A. (2013). Ultrasensitive fluorescent proteins for imaging neuronal activity. Nature.

[38] Grienberger C., Konnerth A. (2012). Imaging calcium in neurons. Neuron.

[39] Plant T.D., Standen N.B., Ward T.A. (1983). The effects of injection of calcium ions and calcium chelators on calcium channel inactivation in Helix neurones. J Physiol.

[40] Yamada Y., Michikawa T., Hashimoto M., Horikawa K., Nagai T., Miyawaki A. (2011). Quantitative comparison of genetically encoded Ca indicators in cortical pyramidal cells and cerebellar Purkinje cells. Front Cell Neurosci.

[41] Yang H.H., St-Pierre F. (2016). Genetically encoded voltage indicators: opportunities and challenges. J Neurosci.

[42] Cohen L.B., Salzberg B.M., Davila H.V., Ross W.N., Landowne D., Waggoner A.S. (1974). Changes in axon fluorescence during activity: molecular probes of membrane potential. J Membr Biol.

[43] Tasaki I., Watanabe A., Sandlin R., Carnay L. (1968). Changes in fluorescence, turbidity, and birefringence associated with nerve excitation. Proc Natl Acad Sci U S A.

[44] Wahl P., Kasai M., Changeux P. (1971). A study on the motion of proteins in excitable membrane fragments by nanosecond fluorescence polarization spectroscopy. Eur J Biochem.

[45] Platt J.R. (1961). Electrochromism, a possible change of color producible in dyes by an electric field. J Chem Phys.

[46] Bücher H., Wiegand J., Snavely B.B., Beck K.H., Kuhn H. (1969). Electric field induced changes in the optical absorption of a merocyanine dye. Chem Phys Lett.

[47] Reich R., Schmidt S. (1972). Über den Einfluß elektrischer Felder auf das Absorptionsspektrum von Farbstoffmolekülen in Lipidschichten. I. Theorie. Berichte Der Bunsengesellschaft Für Phys Chemie.

[48] Loew L.M., Simpson L.L. (1981). Charge-shift probes of membrane potential: a probable electrochromic mechanism for p-aminostyrylpyridinium probes on a hemispherical lipid bilayer. Biophys J.

[49] Loew L.M., Bonneville G.W., Surow J. (1978). Charge shift optical probes of membrane potential. Theory Biochemistry.

[50] Loew L.M., Scully S., Simpson L., Waggoner A.S. (1979). Evidence for a charge-shift electrochromic mechanism in a probe of membrane potential. Nature.

[51] Liptay W. (1956). Die Lösungsmittelabhängigkeit der Wellenzahl von Elektronenbanden und die chemisch-physikalischen Grundlagen. Z Naturforsch.

[52] Hübener G., Lambacher A., Fromherz P. (2003). Anellated hemicyanine dyes with large symmetrical solvatochromism of absorption and fluorescence. J Phys Chem B.

[53] Grinvald A., Fine A., Farber I.C., Hildesheim R. (1983). Fluorescence monitoring of electrical responses from small neurons and their processes. Biophys J.

[54] Grinvald A., Hildesheim R., Farber I.C., Anglister L. (1982). Improved fluorescent probes for the measurement of rapid changes in membrane potential. Biophys J.

[55] Hassner A., Birnbaum D., Loew L.M. (1984). Charge-shift probes of membrane potential. Syn J Org Chem.

[56] Loew L.M., Cohen L.B., Dix J., Fluhler E.N., Montana V., Salama G. (1992). A naphthyl analog of the aminostyryl pyridinium class of potentiometric membrane dyes shows consistent sensitivity in a variety of tissue, cell, and model membrane preparations. J Membr Biol.

[57] Kuhn B., Fromherz P. (2003). Anellated hemicyanine dyes in a neuron membrane: molecular Stark effect and optical voltage recording. J Phys Chem B.

[58] Fromherz P., Hübener G., Kuhn B., Hinner M.J. (2008). ANNINE-6plus, a voltage-sensitive dye with good solubility, strong membrane binding and high sensitivity. Eur Biophys J.

[59] Clarke R.J., Zouni A., Holzwarth J.F. (1995). Voltage sensitivity of the fluorescent probe RH421 in a model membrane system. Biophys J.

[60] González J.E., Tsien R.Y. (1995). Voltage sensing by fluorescence resonance energy transfer in single cells. Biophys J.

[61] Wong K.F., Bagchi B., Rossky P.J. (2004). Distance and orientation dependence of excitation transfer rates in conjugated systems: beyond the Forster theory. J Phys Chem A.

[62] Wu P., Brand L. (1994). Resonance energy transfer: methods and applications. Anal Biochem.

[63] González J.E., Oades K., Leychkis Y., Harootunian A., Negulescu P.A. (1999). Cell-based assays and instrumentation for screening ion-channel targets. Drug Discov Today.

[64] González J.E., Maher M.P. (2002). Cellular fluorescent indicators and voltage/ion probe reader (VIPR): tools for ion channel and receptor drug discovery. Recept Channel.

[65] Chanda B., Blunck R., Faria L.C., Schweizer F.E., Mody I., Bezanilla F. (2005). A hybrid approach to measuring electrical activity in genetically specified neurons. Nat Neurosci.

[66] Li L.S. (2007). Fluorescence probes for membrane potentials based on mesoscopic electron transfer. Nano Lett.

[67] Miller E.W., Lin J.Y., Frady E.P., Steinbach P.A., Kristan W.B. J.r., Tsien R.Y. (2012). Optically monitoring voltage in neurons by photo-induced electron transfer through molecular wires. Proc Natl Acad Sci U S A.

[68] Liu P., Miller E.W. (2020). Electrophysiology, unplugged: imaging membrane potential with fluorescent indicators. Acc Chem Res.

[69] Davis W.B., Svec W.A., Ratner M.A., Wasielewski M.R. (1998). Molecular-wire behaviour in p-phenylenevinylene oligomers. Nature.

[70] Murata Y., Iwasaki H., Sasaki M., Inaba K., Okamura Y. (2005). Phosphoinositide phosphatase activity coupled to an intrinsic voltage sensor. Nature.

[71] Kralj J.M., Douglass A.D., Hochbaum D.R., Maclaurin D., Cohen A.E. (2011). Optical recording of action potentials in mammalian neurons using a microbial rhodopsin. Nat Methods.

[72] Kralj J.M., Hochbaum D.R., Douglass A.D., Cohen A.E. (2011). Electrical spiking in Escherichia coli probed with a fluorescent voltage-indicating protein. Science.

[73] Gong Y., Huang C., Li J.Z., Grewe B.F., Zhang Y., Eismann S. (2015). High-speed recording of neural spikes in awake mice and flies with a fluorescent voltage sensor. Science.

[74] Knöpfel T., Díez-García J., Akemann W. (2006). Optical probing of neuronal circuit dynamics: genetically encoded versus classical fluorescent sensors. Trends Neurosci.

[75] Mutoh H., Akemann W., Knöpfel T. (2012). Genetically engineered fluorescent voltage reporters. ACS Chem Neurosci.

[76] St-Pierre F., Chavarha M., Lin M.Z. (2015). Designs and sensing mechanisms of genetically encoded fluorescent voltage indicators. Curr Opin Chem Biol.

[77] Abdelfattah A.S., Kawashima T., Singh A., Novak O., Liu H., Shuai Y. (2019). Bright and photostable chemigenetic indicators for extended in vivo voltage imaging. Science.

[78] Lin M.Z., Schnitzer M.J. (2016). Genetically encoded indicators of neuronal activity. Nat Neurosci.

[79] Jin L., Han Z., Platisa J., Wooltorton J.R., Cohen L.B., Pieribone V.A. (2012). Single action potentials and subthreshold electrical events imaged in neurons with a fluorescent protein voltage probe. Neuron.

[80] Reeve J.E., Anderson H.L., Clays K. (2010). Dyes for biological second harmonic generation imaging. Phys Chem Chem Phys.

[81] Khadria A., de Coene Y., Gawel P., Roche C., Clays K., Anderson H.L. (2017). Push-pull pyropheophorbides for nonlinear optical imaging. Org Biomol Chem.

[82] Huang J.Y., Lewis A., Loew L. (1988). Nonlinear optical properties of potential sensitive styryl dyes. Biophys J.

[83] Ben-oren I., Peleg G., Lewis A., Minke B., Loew L. (1996). Infrared nonlinear optical measurements of membrane potential in photoreceptor cells. Biophys J.

[84] Clays K., Persoons A. (1991). Hyper-Rayleigh scattering in solution. Phys Rev Lett.

[85] Reeve J.E., Corbett A.D., Boczarow I., Kaluza W., Barford W., Bayley H. (2013). Porphyrins for probing electrical potential across lipid bilayer membranes by second harmonic generation. Angew Chem Int Ed Engl.

[86] Bouevitch O., Lewis A., Pinevsky I., Wuskell J.P., Loew L.M. (1993). Probing membrane potential with nonlinear optics. Biophys J.

[87] Nuriya M., Fukushima S., Momotake A., Shinotsuka T., Yasui M., Arai T. (2016). Multimodal two-photon imaging using a second harmonic generation-specific dye. Nat Commun.

[88] Sacconi L., Dombeck D.A., Webb W.W. (2006). Overcoming photodamage in second-harmonic generation microscopy: real-time optical recording of neuronal action potentials. Proc Natl Acad Sci U S A.

[89] Khadria A., Fleischhauer J., Boczarow I., Wilkinson J.D., Kohl M.M., Anderson H.L. (2018). Porphyrin dyes for nonlinear optical imaging of live cells. iScience.

[90] Reeve J.E., Collins H.A., De Mey K., Kohl M.M., Thorley K.J., Paulsen O. (2009). Amphiphilic porphyrins for second harmonic generation imaging. J Am Chem Soc.

[91] Byrnes K.R., Waynant R.W., Ilev I.K., Wu X., Barna L., Smith K. (2005). Light promotes regeneration and functional recovery and alters the immune response after spinal cord injury. Laser Surg Med.

[92] Lakowicz J.R. (2007).

[93] Kearns D.R. (1971). Physical and chemical properties of singlet molecular oxygen. Chem Rev.

[94] Diaspro A., Chirico G., Usai C., Ramoino P., Dobrucki J. (2006). Photobleaching. In: Pawley JB editors. Handbook of Biological Confocal Microscopy, Boston, MA: Springer.

[95] Huang Y.L., Walker A.S., Miller E.W. (2015). A photostable silicon rhodamine platform for optical voltage sensing. J Am Chem Soc.

[96] Liebmann J., Born M., Kolb-Bachofen V. (2010). Blue-light irradiation regulates proliferation and differentiation in human skin cells. J Invest Dermatol.

[97] Wang L.V., Wu H.I. (2009).

[98] Miyamoto D., Murayama M. (2016). The fiber-optic imaging and manipulation of neural activity during animal behavior. Neurosci Res.

[99] Rao B., Zhang R., Li L., Shao J.Y., Wang L.V. (2017). Photoacoustic imaging of voltage responses beyond the optical diffusion limit. Sci Rep.

[100] Gottschalk S., Degtyaruk O., Mc Larney B., Rebling J., Hutter M.A., Deán-Ben X.L. (2019). Rapid volumetric optoacoustic imaging of neural dynamics across the mouse brain. Nat Biomed Eng.

